# Discordant Results Obtained with *Francisella tularensis* during In Vitro and In Vivo Immunological Studies Are Attributable to Compromised Bacterial Structural Integrity

**DOI:** 10.1371/journal.pone.0058513

**Published:** 2013-03-12

**Authors:** Anju Singh, Tabassum Rahman, Meenakshi Malik, Anthony J. Hickey, Cynthia A. Leifer, Karsten R. O. Hazlett, Timothy J. Sellati

**Affiliations:** 1 Center for Immunology and Microbial Disease, Albany Medical College, Albany, New York, United States of America; 2 Department of Microbiology and Immunology, Cornell University College of Veterinary Medicine, Ithaca, New York, United States of America; University of Louisville, United States of America

## Abstract

*Francisella tularensis* (*Ft*) is a highly infectious intracellular pathogen and the causative agent of tularemia. Because *Ft* can be dispersed via small droplet-aerosols and has a very low infectious dose it is characterized as a category A Select Agent of biological warfare. Respiratory infection with the attenuated Live Vaccine Strain (LVS) and the highly virulent SchuS4 strain of *Ft* engenders intense peribronchiolar and perivascular inflammation, but fails to elicit select pro-inflammatory mediators (*e.g.*, TNF, IL-1β, IL-6, IL-12, and IFN-γ) within the first ∼72 h. This *in vivo* finding is discordant with the principally T_H_1-oriented response to *Ft* frequently observed in cell-based studies wherein the aforementioned cytokines are produced. An often overlooked confounding factor in the interpretation of experimental results is the influence of environmental cues on the bacterium's capacity to elicit certain host responses. Herein, we reveal that adaptation of *Ft* to its mammalian host imparts an inability to elicit select pro-inflammatory mediators throughout the course of infection. Furthermore, *in vitro* findings that non-host adapted *Ft* elicits such a response from host cells reflect aberrant recognition of the DNA of structurally-compromised bacteria by AIM2-dependent and -independent host cell cytosolic DNA sensors. Growth of *Ft* in Muller-Hinton Broth or on Muller-Hinton-based chocolate agar plates or genetic mutation of *Ft* was found to compromise the structural integrity of the bacterium thus rendering it capable of aberrantly eliciting pro-inflammatory mediators (*e.g.*, TNF, IL-1β, IL-6, IL-12, and IFN-γ). Our studies highlight the profound impact of different growth conditions on host cell response to infection and demonstrate that not all *in vitro*-derived findings may be relevant to tularemia pathogenesis in the mammalian host. Rational development of a vaccine and immunotherapeutics can only proceed from a foundation of knowledge based upon *in vitro* findings that recapitulate those observed during natural infection.

## Introduction

*Francisella tularensis* (*Ft*) subspecies *tularensis* is a gram-negative, facultative, intracellular coccobacillus capable of causing a fatal disease called tularemia. *Ft* exists in two clinically-relevant forms, the European biovar B (*holarctica*) that produces acute though mild self-limiting infections and the more virulent United States biovar A (*tularensis*) which often is associated with pneumonic tularemia and more severe disease. Type A strains of *Ft* are extremely virulent with as few as 10 colony-forming units (CFU) causing a lethal infection in untreated individuals. Early-phase (<72 h) murine respiratory infection with either an attenuated Type B Live Vaccine Strain (LVS) or the highly virulent Type A SchuS4 strain is characterized by exponential bacterial replication within the cytosol of host cells without elicitation of select pro-inflammatory cytokines (*i.e.*, TNF, IL-1β, IL-6, IL-12, and IFN-γ) [1]. Not until ≥72 h post-infection do the aforementioned pro-inflammatory molecules become an element of the cytokine ‘storm’ associated with severe sepsis, which culminates in organ failure and death.

Considerable research effort has focused on the use of both *in vitro* and *in vivo* assay systems to reveal the mechanisms by which *francisellae* trigger host cytokine/chemokine production, avoid killing and clearance, and, ultimately, cause death of the host. Various groups, including our own, have shown that the *in vitro* pro-inflammatory response of host cells to *Ft* is Toll-Like Receptor 2 (TLR2)-dependent [2]–[4]. In a C57BL/6 mouse model of respiratory tularemia initiated by infection with *Ft* LVS, the absence of TLR2 engenders accelerated and greater mortality, higher bacterial burden, and dysregulated cytokine production [5], [6]. Experiments using SchuS4 are precluded by the lack of an LD_50_ for such highly virulent Type A strains. TLR2 signaling also is a critical regulator of IFN-γ production in the liver of mice infected with *Ft* [6]. *Ft* possesses TLR2 agonists in the form of lipoproteins (*e.g.*, Tul4 and FTT1103) that stimulate cytokine production in human and mouse cells [7]. Cole *et al.* reported that coordinated engagement of multiple pattern recognition receptors (PRRs) including TLR2, cytosolic sensors, and inflammasome activation by *Ft* are required to elicit host pro-inflammatory responses [2], [8].

Although TLRs are the most extensively characterized of the PRRs responsible for recognition of microbe-associated molecular patterns (MAMPs), studies have identified another mammalian PRR family that senses and responds to pathogens such as *Ft*, which access and replicate within the cytosol [8]. The nucleotide-binding oligomerization domain (NOD)-like receptor proteins (NLRPs) comprise a family of cytoplasmic molecules that interact with the adapter protein apoptosis-associated speck-like protein containing a CARD (ASC). ASC plays a central role in a multiprotein complex known as an inflammasome that promotes the maturation of IL-1β and IL-18 by virtue of its ability to ‘link’ the sensor function of NLRPs with caspase-1, a downstream effector enzyme. Caspase-1 cleaves the pro-forms of IL-1β and IL-18 thus facilitating their secretion from activated cells. Caspase-1 also is involved in certain forms of host cell death. A member of the PYHIN protein family, absent in melanoma 2 (AIM2), also was shown to associate with ASC and be necessary for inflammasome activation and caspase-1-mediated maturation of IL-1β and IL-18 [9]. AIM2 mediates this effect through recognition of dsDNA in the cytosol and recently was reported to play a role in cellular responses to as well as host defense against *Ft* LVS [10], [11] and *F. novicida* [12], [13].

While *Ft* grown in Mueller-Hinton II broth (MHB) or on MH-based chocolate agar plates exhibits a capacity to elicit select pro-inflammatory cytokines (*i.e.*, TNF, IL-1β, IL-6, IL-12, and IFN-γ) from a variety of cell-types within 24 h of *in vitro* infection, such T_H_1-oriented molecules are absent during the first 72 h of *in vivo* infection [5], [14], [15]. Hazlett, *et al.* [16] and others [17]–[20] have reported that *in vitro* growth conditions have a profound qualitative and quantitative effect on the *in vitro* as well as *in vivo* host response to *Ft* LVS and SchuS4. *Ft* cultivated under conditions that preclude host-adaptation (*e.g.*, growth in MHB or on MH-based agar medium) versus those that facilitate host-adaptation [*e.g.*, growth in Brain Heart Infusion broth (BHIB) or replication within isolated MΦ or infected tissues] differs substantially; the former growth conditions impose upon the bacterium a pro-inflammatory phenotype (*i.e.*, the ability to elicit TNF, IL-1β, IL-6, IL-12, and IFN-γ) that *Ft* fails to exhibit *in vivo* during natural infection [21]. Through *in vitro* media-swapping experiments it has been shown that the process of host-adaptation occurs between 12–16 h after switching MHB-grown *Ft* to BHIB [16]. Thus, even when murine infection is initiated with MHB-grown *Ft* the inflammatory response to actively replicating (now, host-adapted) bacteria at 24 h fails to include TNF, IL-1β, and IL-6.

The present study was initiated to investigate the mechanism underlying this observed dichotomy between *in vitro* and *in vivo* host responses to *Ft*. Herein, we report that when host adapted (HAd) to its mammalian environment through *in vitro* cultivation in BHIB, recovery from infected MΦ, or growth within infected tissues, *Ft* LVS as well as SchuS4 is incapable of eliciting select pro-inflammatory cytokines from host cells either *in vitro* or *in vivo*. The robust induction of TNF, IL-1β, and IL-6 observed *in vitro* following incubation with MHB-grown *Ft* or HAd-*Ft* mutants reflects the compromised structural integrity of the bacteria. This aberrant *in vitro* inflammatory response, which is absent *in vivo*, is driven by AIM2-dependent and -independent sensing of bacterial DNA associated with damaged *Ft*.

## Results

### CD14 and TLR2 deficiency enhances susceptibility to disease following infection with *Ft* LVS

**Figure 1:**
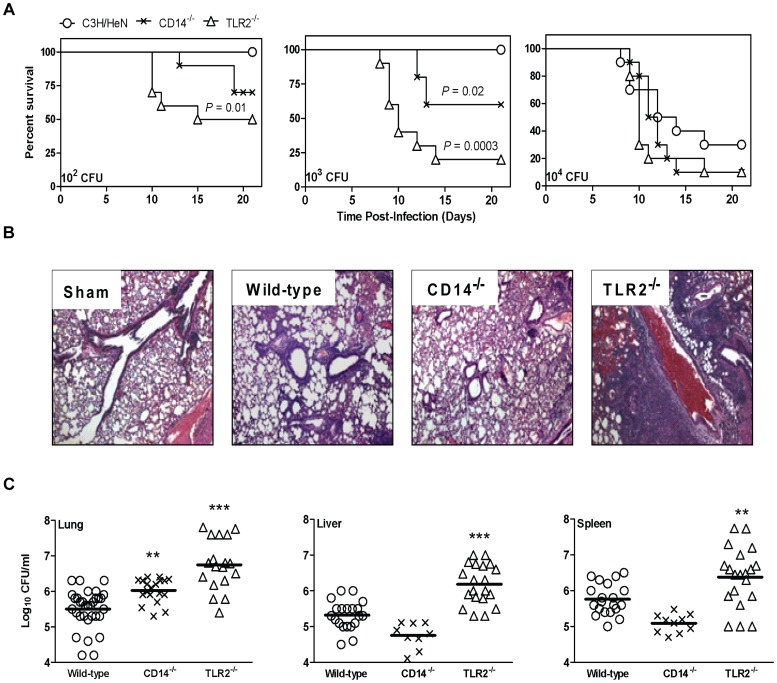
CD14 and TLR2 deficiency impairs host immunity to respiratory challenge with *Francisella tularensis* (*Ft*). (A) Wild-type (C3H/HeN) mice and their CD14^−/−^ and TLR2^−/−^ counterparts were challenged intranasally (i.n.) with *Ft* LVS ranging in dosage from 10^2^ to 10^4^ colony forming units (CFU) and were monitored for survival. Results are expressed as Kaplan-Meier curves and *P* values determined by log rank test. The results show pooled data of two independent experiments (n = 6 mice per group or 12 mice total). (B) Histopathological changes in the lungs of C3H/HeN mice and the respective mutant counterparts were evaluated 7 days post i.n. infection (p.i.) with 10^3^ CFU of *Ft* LVS. Lung sections of sham-inoculated mice served as a control. Magnification 100×. (C) C3H/HeN and the respective mutant counterparts were inoculated i.n. with 10^3^ CFU of *Ft* LVS. At day 7 post-infection, mice were sacrificed and homogenates of the lungs, liver, and spleen were plated for determination of bacterial burden. Results represent the mean ± SEM of CFU counts from two to three independent experiments (n = 6 mice per group or 12 to 18 mice total). (All results shown were subjected to One-way ANOVA with Bonferroni's Post-test, *P<0.05, **P<0.01, ***P<0.001).

To confirm and extend our previous findings using a C57BL/6 mouse model of respiratory tularemia [5] the role of PRRs was evaluated in C3H/HeN, CD14^−/−^, and TLR2^−/−^ mice that were inoculated intranasally (i.n.) with *Ft* LVS grown in MHB. The rationale for studying the role of CD14 in modulating host cell response to *Ft* is that this co-receptor facilitates recognition of a variety of MAMPs by TLRs; in particular, bacterial lipoproteins by TLR2 [22]. To assess relative susceptibility to infection, genotypes were compared based on the cumulative proportion of animals in a group that survived following inoculation and the group's median time to death (MTD). As seen in Figure 1A, infection of mice with as few as 10^2^ CFU resulted in increased mortality in CD14^−/−^ and TLR2^−/−^ mice, though only the latter reached statistical significance wherein the MTD was 18 days versus >21 and the percentage surviving was 50% versus 100% for control animals. With a 10^3^ CFU inoculum the MTD for CD14^−/−^ and TLR2^−/−^ mice was >21 days and 10 days, respectively. Only 60% of the CD14^−/−^ and 20% of TLR2^−/−^ mice survived when compared to 100% survival of the wild-type group. Differences in susceptibility were masked at a 10^4^ CFU challenge due to the virulence of the pathogen (Figure 1A).

To determine whether PRR^−/−^ mice exhibit distinct histopathological changes compared to wild-type animals we evaluated the lungs of animals at 7 days post-i.n. inoculation (p.i.) with 10^3^ CFU. Peribronchiolar inflammation and parenchymal pneumonia was assessed as described previously [5]. Compared with sham inoculated mice, infected mice presented with pneumonic inflammatory infiltrates consisting of intra-alveolar and interstitial neutrophils, variable amounts of MΦ, and focal necrosis found within loose connective tissue (Figure 1B). The pneumonia typically involved one or more lobes and extended to the pleura. Marked differences were observed with wild-type mice showing a predominantly mild pneumonia with mild to moderate peribronchiolar inflammation, which was more organizing than necrotizing when compared to the PRR^−/−^ mice. TLR2^−/−^ mice presented with more severe pneumonia having both organizing and necrotizing features involving multiple lobes of the lung and moderate to severe peribronchiolar inflammation. The luminal spaces of bronchioles in the TLR2^−/−^ mice occasionally contained sloughed lining (*i.e.*, epithelial) cells, degenerated neutrophils and alveolar MΦ; features that were less prominent in wild-type animals. Additionally, the alveolar walls in the lungs of TLR2^−/−^ mice contained mononuclear and fibroblast cell infiltrations and activated alveolar septal cells. By contrast, the CD14^−/−^ mice exhibited pathologic changes that were milder than in TLR2^−/−^ mice, but, greater than in wild-type animals (Figure 1B).

Inability to control bacterial replication as well as clear organisms represents a pathogenic mechanism consistent with greater tissue pathology and increased morbidity and mortality. To explore further this relationship, C3H/HeN, CD14^−/−^, and TLR2^−/−^ mice were i.n. inoculated with 10^3^ CFU of *Ft* LVS and bacterial burden in the lungs, liver, and spleen was determined. At day 7 p.i., bacterial burdens in the lungs of CD14^−/−^ and TLR2^−/−^ mice were significantly higher than in wild-type mice (Figure 1C). TLR2^−/−^ mice, but not CD14^−/−^ mice, had significantly higher bacterial burden in liver and spleen as well. *In vitro* infection of mouse bone marrow-derived monocytes (BMDMs) with *Ft* revealed that by 24 h of co-incubation ∼1.5-log more total bacteria (intra- and extracellular combined) were recovered from TLR2^−/−^, but not CD14^−/−^, cells compared to wild-type cells (data not shown). This impact of TLR2 deficiency on intracellular bacterial replication is consistent with previous findings using BMDMs from C57BL/6 mice [5].

### Cellular responsiveness to *Ft* LVS and SchuS4 *in vitro* is conferred principally by TLR2

**Figure 2:**
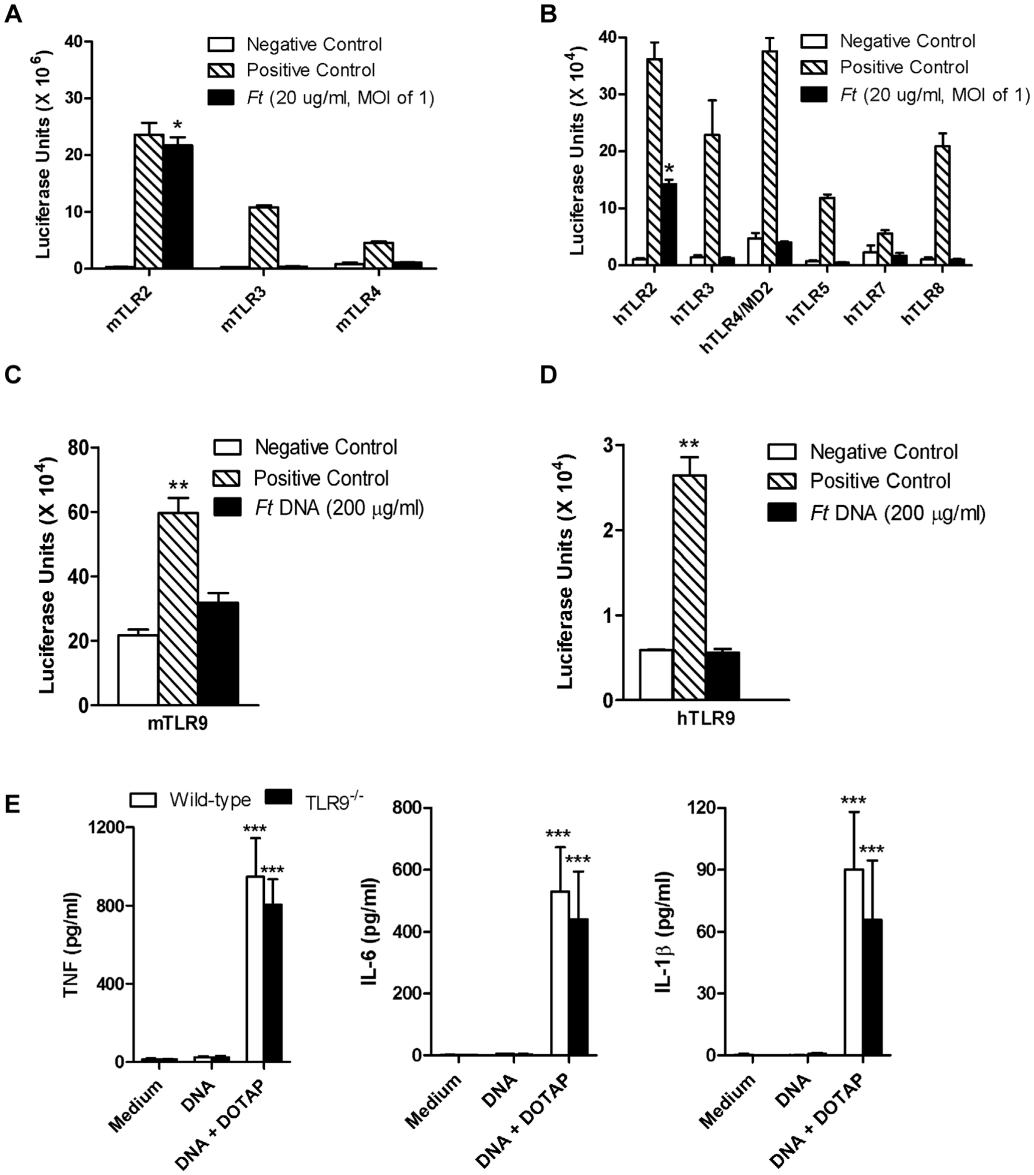
Cellular responsiveness to *Ft* is conferred principally by TLR2. (A) HEK 293 cells stably transfected to express individual members of the mouse TLR family were incubated with a whole cell lysate of *Ft* LVS or a TLR-specific agonist as a control. The extent of cellular responsiveness after 24 h is presented in the form of relative luciferase units. *P<0.05. (B) Similar results were obtained using HEK 293 cells transfected with human TLR homologues. *P<0.05. Control agonists used in (A) and (B) include 1 µg/ml Pam_3_Cys (TLR2), 1 µg/ml poly(I∶C) (TLR3), 100 ng/ml flagellin (TLR5), 100 ng/ml LPS (TLR4), and 100 µM loxoribine (TLR7/8). HEK 293 cells stably transfected with mouse (C) and human (D) TLR9 were incubated with either 20 or 200 µg/ml of genomic DNA purified from *Ft* LVS or unmethylated CpG DNA as a positive control. Cellular responses measured after 24 h are presented in the form of relative luciferase units. **P<0.01. (E) Bone marrow-derived macrophages (BMDMs) (2.5×10^5^ cells/well) from wild-type (C57BL/6) and TLR9^−/−^ mice were incubated with 8 µg/ml of genomic DNA purified from *Ft* LVS either in the absence or presence of DOTAP. The levels of cytokines released after 24 h were determined by Cytometric Bead Array (CBA) or ELISA. Results represent the mean ± SEM from three independent experiments. *** *P*<0.001. (All results shown were subjected to One-way ANOVA with Bonferroni's Post-test).

While TLR2 clearly plays an important role in the host response to *Ft*, other TLRs as well as intracytoplasmic sensors (*e.g.*, AIM2) also may participate in recognition of this pathogen. Using the luciferase reporter assay system, we found that only stable transfection of HEK293 cells with mouse (Figure 2A) and human (Figure 2B) TLR2 conferred responsiveness to MHB-grown *Ft*. As little as one bacterium per 10 cells or the equivalent of 20 µg of total *francisellae* protein per ml was able to significantly increase luciferase activity. We also tested whether purified *Ft* DNA could stimulate cells via TLR9. TLR9 is expressed intracellularly, within the endosomal compartment, and functions to alert the immune system to the presence of bacteria whose DNA, unlike that of eukaryotes, is rich in unmethylated CpG motifs. Despite incubation with as much as 200 µg/ml of *Ft* LVS DNA, HEK293 cells expressing mouse (Figure 2C) or human (Figure 2D) TLR9 failed to respond. Similar results were obtained using DNA (200 µg/ml) isolated from *Ft* SchuS4 (data not shown). Interestingly, as little as 8 µg/ml of *Ft* LVS DNA (Figure 2E) or *Ft* SchuS4 DNA (data not shown) could elicit TNF, IL-1β, and IL-6 from wild-type and TLR9^−/−^ BMDMs; but only when added along with DOTAP, a liposomal transfection reagent. DOTAP carries DNA into the endocytic pathway, but also into the cytosol [23]. Consistent with results in Figure 2C and 2D, there was no difference in cytokine production by BMDMs from wild-type or TLR9^−/−^ C57BL/6 mice following 24 h co-incubation with *Ft* DNA+DOTAP (Figure 2E) or MHB-grown *Ft* (data not shown).

### AIM2-dependent and -independent recognition of *Ft* DNA and non-HAd-*Ft* elicits an aberrant pro-inflammatory cytokine response

**Figure 3:**
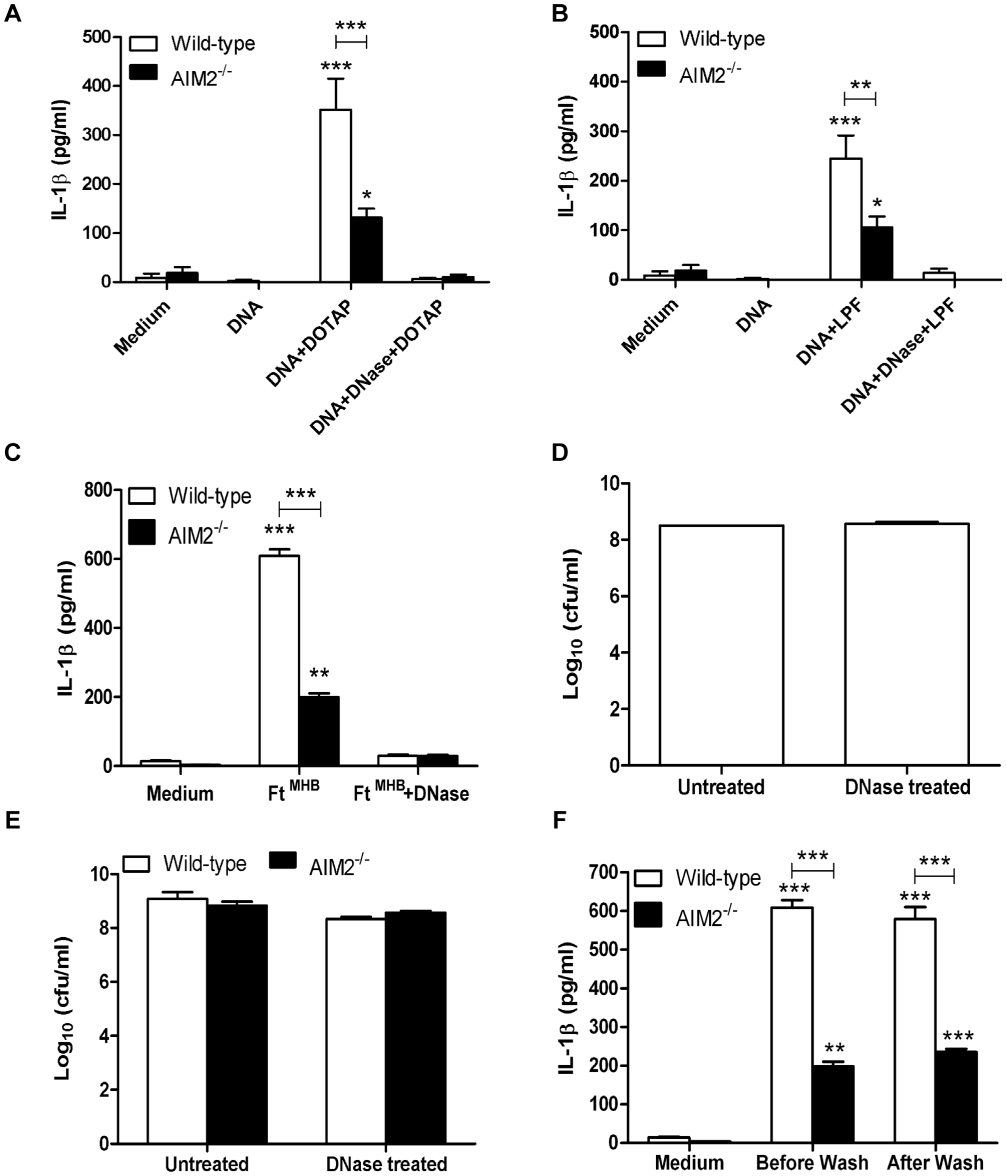
DNase I treatment of DNA and non-HAd *Ft* completely ablates the pro-inflammatory cytokine response. (A) Wild-type and AIM2^−/−^ BMDMs (2.5×10^5^ cells/well) were incubated either in the absence or presence of DOTAP and 8 µg/ml of genomic DNA purified from *Ft* LVS. DNA was either untreated or incubated with DNase I as described in Methods. The levels of the cytokines released after 24 h were determined by CBA or ELISA. ***P<0.001. (B) Similar experiments as in (A) were conducted using Lipofectamine™ 2000 as the transfection agent. *P<0.05, **P<0.01, ***P<0.001. (C) Wild-type and AIM2^−/−^ BMDMs were infected with either untreated or DNase I treated non-HAd bacteria at a MOI of 100 as described in Methods. The supernatants were collected after 24 h and analyzed for the presence of cytokines. **P<0.01, ***P<0.001. (D) The viability of the bacteria before and after DNase I treatment was determined by colony plating. (E) Wild-type and AIM2^−/−^ BMDMs were analyzed for their ability to support bacterial replication and bacterial burden was quantified after 24 h. (F) Wild-type and AIM2^−/−^ BMDMs were infected with non-HAd *Ft* at a MOI of 100 with or without prior washing of the bacteria in cell culture medium. **P<0.01, ***P<0.001. (All results shown were subjected to One-way ANOVA with Bonferroni's Post-test).

Given reports that AIM2 mediates cellular responses to different *Francisella* species [10], [11], [13], the following experiments were undertaken to confirm and extend these findings. Using immortalized wild-type and AIM2^−/−^ BMDMs, *Ft* DNA was incapable of eliciting IL-1β secretion unless coupled with DOTAP (Figure 3A). AIM2 deficiency significantly reduced cytokine release. *Ft* DNA also had a much smaller, yet reproducible, capacity to elicit IL-1β in an AIM2-independent fashion suggesting the existence of another DNA sensor(s) (Figure 3A). Both the AIM2-dependent and -independent production of IL-1β was completely ablated if *Ft* DNA was digested using DNase I. When the liposomal transfection reagent Lipofectamine™ 2000 was used instead of DOTAP essentially the same results were obtained (Figure 3B). Importantly, neither transfection reagent alone elicited a cytokine response from host cells (data not shown). AIM2 also regulated TNF and IL-6 production (Figures S1A and S1B, respectively) though the amplitude of the response and the reliance on AIM2 differed depending on the transfection reagent used (Figures S1A versus 1C and 1B versus 1D). To our knowledge, this is the first report that AIM2 signaling affects the production of pro-inflammatory cytokines such as TNF and IL-6 in addition to IL-1β. Next, it was determined whether MHB-grown *Ft* could elicit IL-1β in an AIM2-dependent as well as -independent manner. As seen in Figure 3C, infection with *Ft* triggered a predominantly AIM2-dependent IL-1β response. Unexpectedly, IL-1β production by the infected BMDMs was eliminated if bacteria were exposed to DNase I prior to co-incubation with cells. This loss of IL-1β-stimulatory activity was neither attributable to an effect of DNase I on bacterial viability (Figure 3D) nor differences in the ability of untreated or DNase I-treated *Ft* to successfully replicate within wild-type and AIM2^−/−^ BMDMs (Figure 3E). The possibility that bacteria were actively secreting or releasing any proteins or DNA into the medium that could account for the level of IL-1β observed also was ruled out. If such were the case, the stimulatory capacity of *Ft* would have been altered by washing the bacteria prior to their co-incubation with BMDMs (Figure 3F). MHB-grown *Ft* also was capable of eliciting TNF and IL-6 in an AIM2-dependent as well as -independent manner, which was partly eliminated in the presence of DNase I (Figures S1E & S1F respectively).

### *In vitro* cell-based and *in vivo* infection studies provide a dichotomous picture of whether *Ft* can directly stimulate the release of select pro-inflammatory cytokines

**Figure 4:**
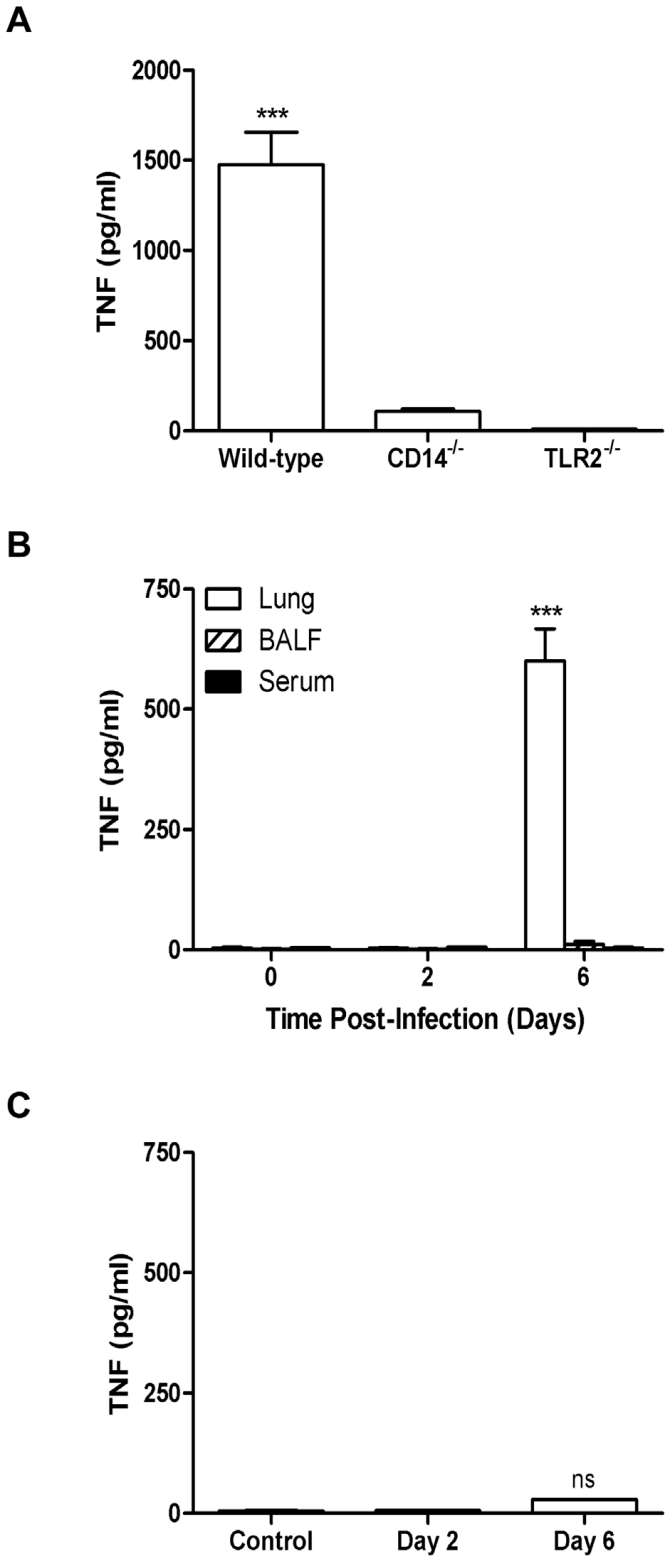
Respiratory tularemia is characterized by an absence of T_H_1-type pro-inflammatory cytokines during early-phase of disease. (A) BMDMs (2.5×10^5^ cells/well) from wild-type (C57BL/6) mice and their mutant counterparts were infected with MHB-grown *Ft* LVS at a MOI of 100. Supernatants were collected after 24 h and analyzed for the presence of cytokines by CBA. ***P<0.001. (B) Cell-free lung homogenates, BALF, and serum from C57BL/6 mice were recovered at 2 and 6 days p.i. following i.n. administration of 10^3^ CFU of *Ft* LVS and were analyzed for the presence of pro-inflammatory cytokines by ELISA. ***P<0.001. (C) BMDMs (2.5×10^5^ cells/well) were incubated for 24 h with *Ft* isolated from mouse lungs at 2 and 6 days p.i. Supernatants were collected and analyzed for the presence of TNF by ELISA and compared to levels of TNF observed *in vivo* at the same point in time. ***P<0.001, ns = not significant. All cell activation assays were conducted using a MOI of 100. Results represent the mean ± SEM from two independent experiments. (All results shown were subjected to One-way ANOVA with Bonferroni's Post-test).

Co-incubation of wild-type, CD14^−/−^, and TLR2^−/−^ BMDMs with MHB-grown *Ft* LVS demonstrates that the TNF produced within 24 h requires both CD14 and TLR2 signaling (Figure 4A). However, at first blush, a paradox arises when one considers the lack of TNF in lung homogenates, bronchoalveolar lavage fluid (BALF), and serum at day 2 p.i. (Figure 4B). A seminal report by Hazlett *et al.* in 2008 [16] and a follow-up report in 2011 [24] resolves this apparent contradiction by showing that *in vitro* growth conditions have a profound qualitative and quantitative effect on both the *in vitro* and *in vivo* host response to *Ft* LVS and SchuS4. When grown in MHB, a medium which restricts the bacterium's ability to ‘host-adapt’ to its mammalian environment, *Ft* LVS exhibits a pro-inflammatory phenotype that results in aberrant production of TNF, IL-1β, and IL-6. In contrast, when grown in BHIB or harvested after *in vitro* proliferation within MΦ, either of which facilitates host adaptation, *Ft* fails to exhibit this pro-inflammatory phenotype and instead displays a predominantly anti-inflammatory phenotype [21]. HAd-*Ft* LVS and SchuS4 elicit anti-inflammatory cytokines (*e.g.*, IL-10 and TGF-β) and induce the generation and proliferation of tolerogenic dendritic cells (tDCs) and regulatory T cells (T_regs_) [21].

**Figure 5:**
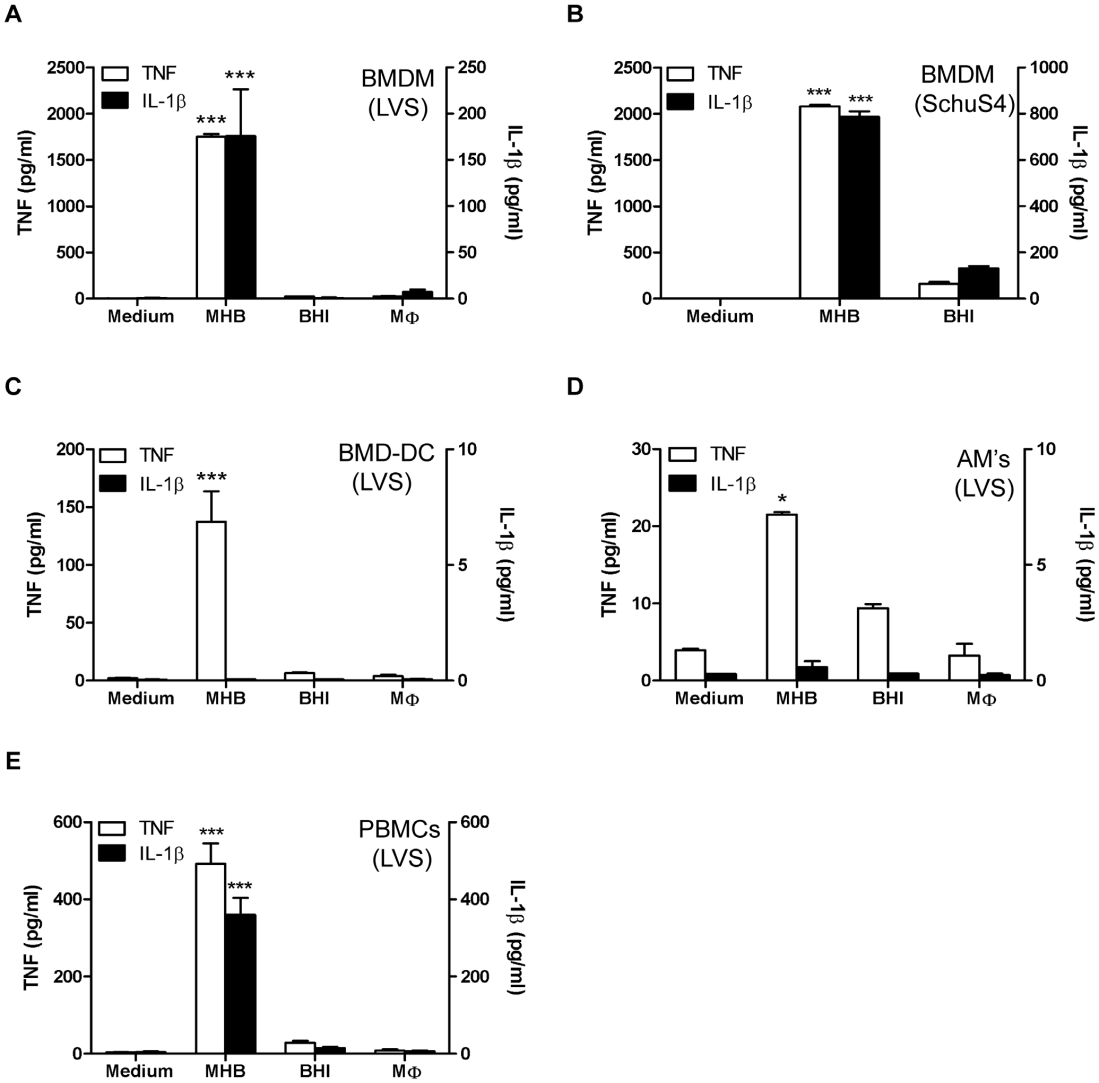
HAd-*Ft* LVS is incapable of eliciting T_H_1-type pro-inflammatory responses irrespective of the cell-type. BMDMs (A & B), BMD-dendritic cells (BMD-DCs) (C), alveolar macrophage MHS cell line (D), and human peripheral blood-derived macrophages (E) were infected at a MOI of 100 with *Ft* grown in MHB or BHIB or bacteria derived from macrophages. Regardless of cell-type, 2.5×10^5^ cells were plated per well, supernatants were collected after 24 h and analyzed for the presence of cytokines by CBA. Results represent the mean ± SEM from two independent experiments. *P<0.05, ***P<0.001. (One-way ANOVA with Bonferroni's Post-test).

Interestingly, despite their absence during early-phase respiratory tularemia, levels of these select pro-inflammatory cytokines (Figures 4B) were significantly elevated by 6 days p.i.. A possible explanation for this finding is that *Ft* replicating during the course of infection acquire an inherent capacity to stimulate such host responses. To explore this possibility, bacteria were recovered from lung tissue at 2 and 6 days p.i. and co-incubated with BMDMs for 24 h. As seen in Figure 4C, bacteria recovered from inflamed lungs at day 6 were incapable of eliciting TNF from BMDMs as is observed in the lungs at this same point in time during natural infection. Also absent were IL-1β, IL-6, and IL-12 (data not shown). Thus, once adapted to the mammalian environment, the inability of *Ft* to directly stimulate select pro-inflammatory cytokines *in vivo* suggests prior *in vitro* studies using bacteria grown in MHB may have engendered results not physiologically-relevant to the disease process. To further test this hypothesis, *Ft* LVS grown in MHB, BHIB, and those isolated from MΦ were used to initiate infection of BMDMs *in vitro*. As seen in the Figure 5A, HAd-*Ft* LVS was incapable of eliciting TNF or IL-1β from BMDMs. Comparable results were obtained using *Ft* LVS and SchuS4 isolated from infected lung, liver, and spleen at day 6 p.i. (data not shown). Importantly, unlike *Ft* SchuS4 grown in BHIB, *Ft* SchuS4 grown in MHB were as pro-inflammatory with regard to stimulating production of TNF or IL-1β (Figure 5B) as their non-HAd-*Ft* LVS counterparts. HAd-*Ft* LVS also failed to elicit such cytokines from mouse BMD-DCs (Figure 5C) or the alveolar MΦ cell line MHS (Figure 5D). Notably, even the non-HAd bacteria failed to stimulate secretion of IL-1β from BMD-DCs or alveolar MΦ. Additionally, primary peripheral blood-derived human monocytes were assayed for the production of cytokines following infection with *Ft* LVS grown in MHB, BHIB, and MΦ. HAd-*Ft* LVS were incapable of eliciting TNF and IL-1β (Figure 5E) or IL-6 (Figure S2A), results also observed using HAd-*Ft* SchuS4 (data not shown). However, HAd-*Ft* did stimulate significant release of IL-8 (Figure S2B) and IL-10 (Figure S2C), but not IFN-γ (Figure S2D) or TGF-β (though a trend towards increased levels was evident, data not shown).

**Figure 6:**
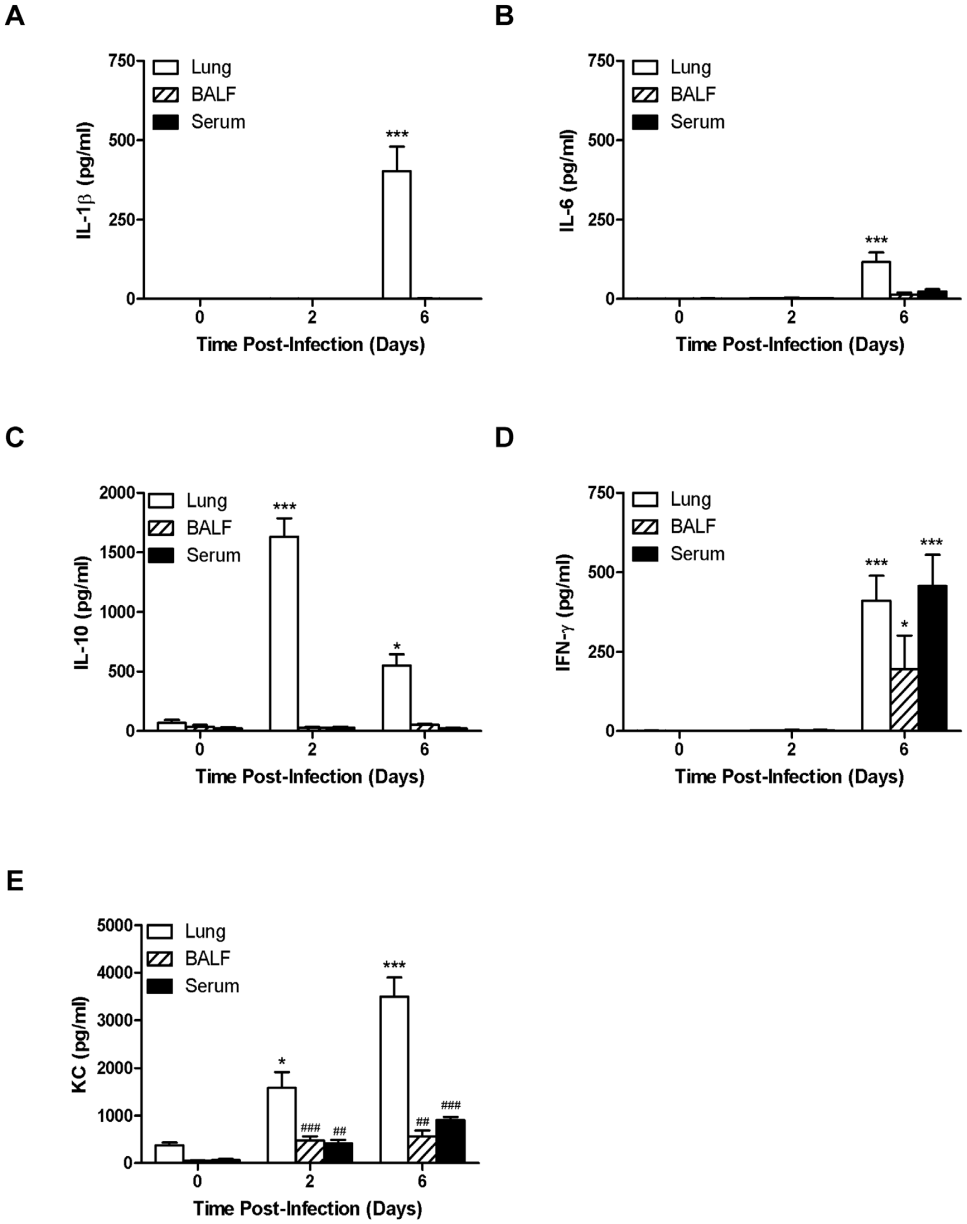
In vivo detection of T_H_1-type cytokines in different anatomic compartments of *Ft* LVS infected mice. Cell-free lung homogenates, BALF and serum from C57BL/6 mice were recovered at 2 and 6 days p.i. following i.n. administration of 10^3^ CFU of *Ft* LVS and were analyzed for the presence of IL-1β (A), IL-6 (B), IL-10 (C), IFN-γ (D), and KC (E) by CBA. *P<0.05, ***P<0.001 by ANOVA and ^##^ *P*<0.0015, ^###^ *P*<0.0008 by Student's t-test.

Next, we sought to more broadly characterize the *in vivo* kinetics of T_H_1-oriented cytokine production in different anatomic compartments by choosing a point during the early- (*i.e.*, day 2) and late-phase (*i.e.*, day 6) of respiratory tularemia and considering day 3 to be a period of transition or tipping point. As was observed for TNF (Figure 4B), while present in the lung at day 6, IL-1β and IL-6 were absent from the BALF and serum and were not present at day 2 (Figures 6A and 6B, respectively). In contrast, production of IL-10 was observed during early- and late-phase tularemia (Figure 6C), consistent with our previous observation of rapid establishment of a principally anti-inflammatory environment in the lungs of *Ft*-infected mice [21]. Differing from the pattern of compartmentalization seen in Figures 4B and 6A–C, IFN-γ was found in tissue, BALF, and serum during late-phase disease; but again, was absent at day 2 (Figure 6D). Finally, levels of KC were significantly elevated in the lungs at days 2 and 6 and to a lesser extent in the BALF and serum (Figure 6E).

**Figure 7:**
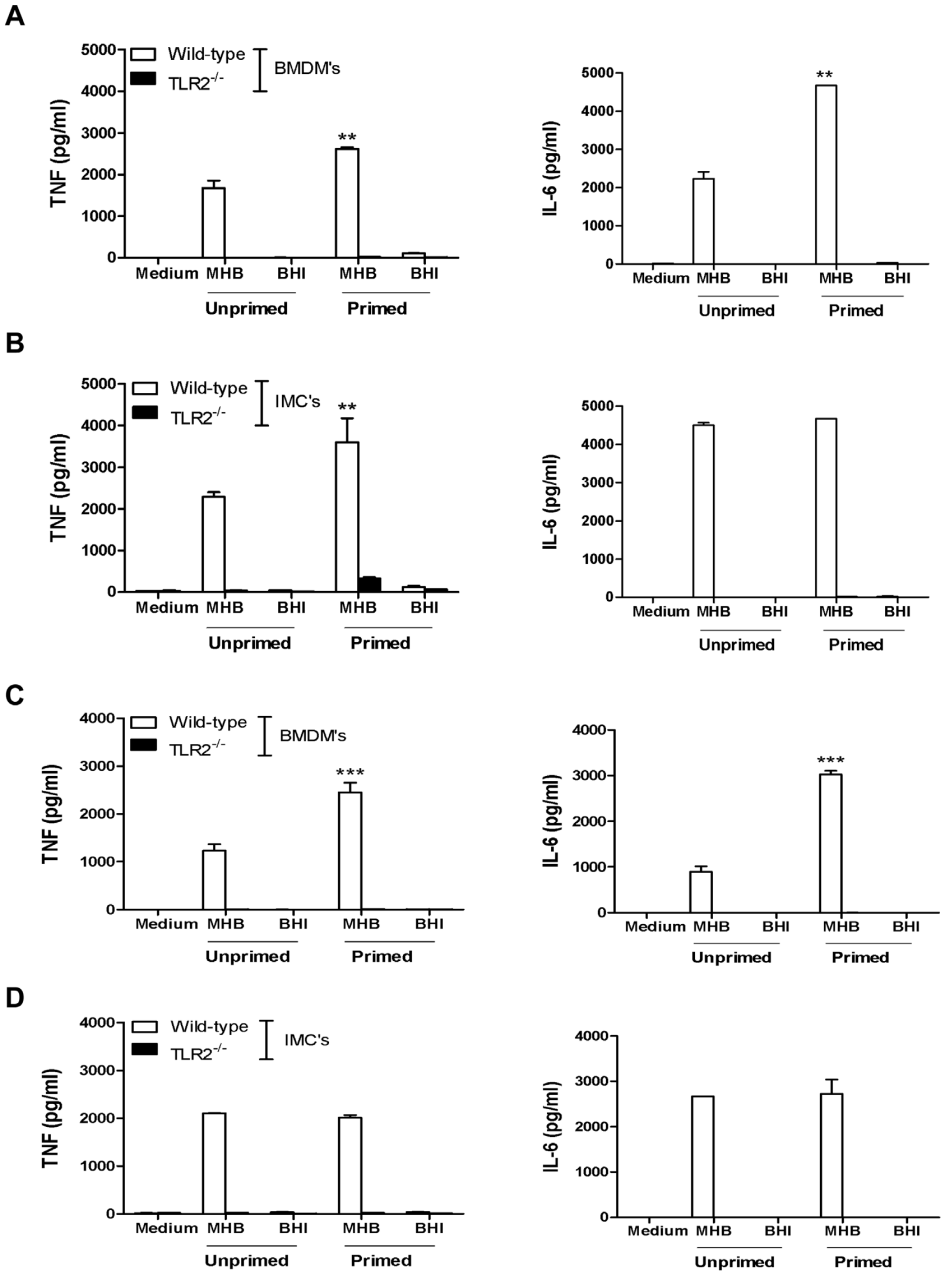
Priming enhances BMDMs responses to non-HAd, but not HAd, *Ft*. (A) Primary BMDMs and (B) immortalized BMDMs (IMC) from wild-type and TLR2^−/−^ C57BL/6 mice (2.5×10^5^ cells/well) were treated with IFN-γ (50 ng/well) for 16 h prior to infection with MHB- and BHI-grown *Ft* LVS at a MOI of 100. (C) In a separate set of experiments priming was achieved by incubation of cells with 4% autologous serum for 16 h prior to infection with *Ft* LVS. The autologous serum was recovered from *Ft* LVS-infected mice at day 6 p.i. For both sets of experiments, supernatants were collected 24 h p.i. and were assayed for the presence of cytokines by CBA. Results represent the mean ± SEM from three independent experiments. **P<0.01, ***P<0.001. (All results shown were subjected to One-way ANOVA with Bonferroni's Post-test).

Given that IFN-γ can prime MΦ to mount a heightened response to bacterial stimuli we revisited the issue of whether HAd-*Ft* is incapable of eliciting select pro-inflammatory cytokines from BMDMs, even when the MΦ are primed. BMDMs either were unprimed or primed with 50 ng of recombinant mouse IFN-γ for 16 h prior to co-incubation with *Ft* grown in MHB or BHIB. At 24 h p.i. both TNF and IL-6 levels were determined and primed BMDMs released significantly more cytokine in response to MHB-grown *Ft* than their unprimed counterparts, a response that was entirely TLR2-dependent (Figure 7A). However, despite exposure to IFN-γ, BMDMs remained unresponsive to HAd-*Ft*. Essentially the same results, with the exception of no change in IL-6 output, were obtained using a primary murine BMDM cell line immortalized via viral transformation (IMC) (Figure 7B). Although IFN-γ priming failed to alter TNF and IL-6 production following infection with HAd-*Ft* it must be acknowledged that the more complex milieu of inflammatory mediators found at day 6 p.i. may be necessary to augment *in vivo* cellular responses to *Ft*. To more faithfully recapitulate this *in vivo* inflammatory environment, recombinant IFN-γ was replaced with 4% autologous serum recovered from wild-type and TLR2^−/−^ mice at day 6 p.i. Following 16 h of pre-exposure to 4% serum, BMDMs were more responsive to MHB-grown *Ft*, but, not to bacteria pre-adapted to their mammalian environment (Figure 7C). Furthermore, unlike with IFN-γ priming, exposure to serum from infected mice did not enhance production of either cytokine by IMC following infection with *Ft* (Figure 7D). Whenever cytokines were released by either primary or immortalized MΦ, this was a TLR2-dependent event (Figures 7C and 7D, respectively).

### Growth of *Ft* LVS in MHB compromises the structural integrity of the bacterium

**Figure 8:**
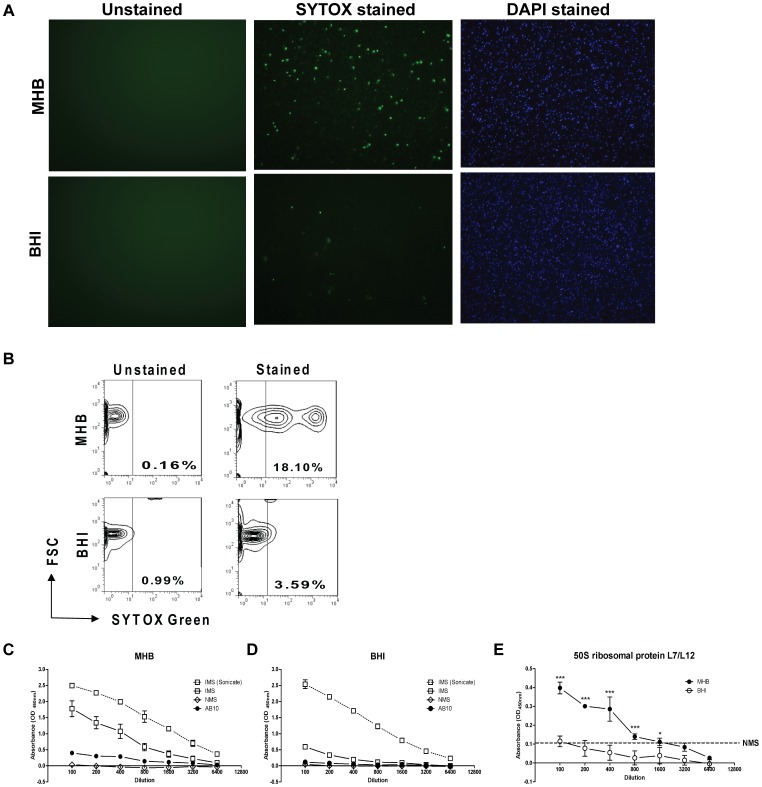
Compared to HAd-*Ft*, non-HAd *Ft* are structurally compromised. (A) The ViaGram™ Red^+^ bacterial Gram Stain and Viability kit was used to stain HAd-adapted and the non-HAd bacteria. *Ft* with intact cell membranes stain fluorescent blue with 4′,6-diamidino-2-phenylindole (DAPI), whereas bacteria with damaged membranes stain fluorescent green with SYTOX Green nucleic acid stain, which displaces the DAPI. *Ft* was visualized using standard fluorescence microscopy. (B) The percentage of structurally compromised bacteria was quantified by flow cytometry. Whole or sonicated (C) non-HAd and (D) HAd-*Ft* (5×10^6^/well) were coated onto a 96-well microtiter plate and these target antigens were probed with immune sera (IMS) recovered from *Ft* LVS-infected mice, normal mouse sera (NMS), and a monoclonal antibody (mAb) (AB10) specific for the *Ft* internal 50S ribosomal protein. Biotinylated anti-mouse IgG mAb was used as a secondary antibody and the plate was developed with TMB substrate (100 µl/well). Reactions were stopped with 2N H_2_SO_4_ and absorbance readings were taken at A_450 nm_. (E) Data for AB10 immune reactivity parsed out from panels (C) and (D) to facilitate comparative analysis. Dotted line indicates limit of detection observed with NMS, which did not differ when antigenic target was MHB- or BHI-grown *Ft*. *P<0.001, ***P<0.001.

The unanticipated ability of DNase I treatment to ablate TNF, IL-1β, and IL-6 production by BMDMs in response to MHB-grown *Ft* raised the question of whether the bacterium's DNA was exposed to host cells. *Ft* grown in either MHB or BHIB to an early log-phase A_600 nm_ optical density of 0.2 were stained using a viability kit that discriminates live from damaged as well as dead organisms on the basis of bacterial membrane integrity and the ability of the SYTOX® Green dye to intercalate nucleic acid. As shown by fluorescence microscopy (Figure 8A) and quantified by flow cytometry (Figure 8B), a much greater proportion of MHB- versus BHIB-grown *Ft* was labeled (18.1% versus 3.59%, respectively). To evaluate further the impact of growth in MHB, an ELISA was established where whole MHB- and BHIB-grown *Ft* served as the antigenic “target” for non-immune mouse serum (NMS), *Ft*-specific immune serum (IMS), or a monoclonal antibody (AB10) directed against FTL1745, the cytoplasmic *Ft* 50S ribosomal protein L7/L12 [25]. Sonicated bacteria served as a control for complete loss of structural integrity. As seen in Figure 8C and 8D, while IMS had an equivalent capacity to react with sonicated *Ft* regardless of growth medium used, immune reactivity to whole MHB-grown bacteria was much greater than to BHIB-grown bacteria. This difference reflects the fact that growth in BHIB causes *Ft* to maintain membrane integrity and to express considerably more capsule thus masking antigenic epitopes expressed on the bacterium's outer membrane and those that are subsurface localized [24]. AB10 showed no greater ability than NMS to bind BHIB-grown *Ft* (Figure 8D). In contrast, AB10 reactivity to MHB-grown *Ft* was significantly greater than that observed with their BHIB-grown counterparts (Figure 8E, results parsed out of Figures 8C and 8D to facilitate comparison). Importantly, expression of FTL1745 in MHB- and BHIB-grown *Ft* is equivalent as determined by proteomic analysis (data not shown). Collectively, these findings are consistent with the DNase I sensitivity results suggesting that growth in MHB structurally compromises the bacterium. Differences in the structural integrity between MHB- and BHIB-grown *Ft* also are evident at the electron microscopic level [16] and the morphological changes MHB-grown *Ft* undergoes mirrors that observed due to mutation of a wide variety of genes of disparate function in *F. novicida* [26].

**Figure 9:**
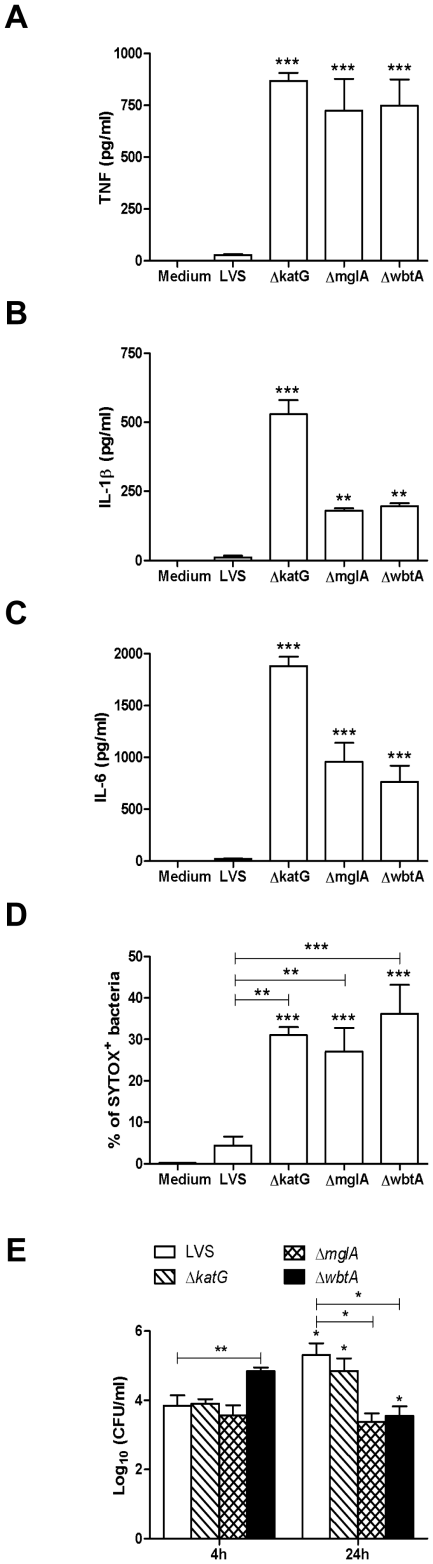
Genetic mutation of HAd-*Ft* confers a pro-inflammatory phenotype upon the bacterium. Primary BMDMs (2.5×10^5^ cells/well) were infected with wild-type and mutant HAd-*Ft* at a MOI of 100. Supernatants were collected 24 h p.i. and were assayed for the presence of (A) TNF, (B) IL-1β, and (C) IL6 by CBA. Results represent the mean ± SEM from four independent experiments. **P<0.01, ***P<0.001. (D) HAd-*Ft* and the corresponding mutants were stained with SYTOX® Green nucleic acid stain and were analyzed for SYTOX^+^ bacteria by flow cytometry. Results represent the mean ± SEM from three independent experiments. **P<0.01, ***P<0.001. (E) Primary BMDMs (2.5×10^5^ cells/well) were infected with wild-type and mutant HAd-*Ft* at a MOI of 100. After gentamycin treatment for 2 h, intra and extracellular bacteria were recovered at 4 and 24 h and enumerated by colony plating. Results represent the mean ± SEM from three independent experiments. *P<0.05, **P<0.01. (All results shown were subjected to One-way ANOVA with Bonferroni's Post-test).

Further support that the general ‘health’ as well as structural integrity of *francisellae* species can alter the nature of the host response is found in an elegant study conducted by Peng *et al.*, 2011 [26]. The impact of genetic mutation of *F. novicida* on engagement of multiple innate immune pathways, including the inflammasome, was shown to be pro-inflammatory in nature as a consequence of a subset of mutant bacteria readily undergoing lysis within the cytosol of infected cells. Release of bacterial components (*e.g.*, DNA) from mutants within the cytosol triggered AIM2-dependent pyroptosis and release of IL-1β. Also produced were levels of TNF and IL-6 far in excess of amounts observed when cells were infected with wild-type *F. novicida*. Similarly, we observed that despite being host-adapted, when *Ft* LVS carries engineered mutations in *katG*, *mglA*, or *wbtA* the bacteria are significantly more pro-inflammatory as determined by release of TNF (Figure 9A), IL-1β (Figure 9B), and IL-6 (Figure 9C). Consistent with this finding was the observation that a higher percentage of mutants were positive for SYTOX® Green dye staining implying compromised membrane integrity (Figure 9D). Similar observations have been made using equivalent SchuS4 mutants (data not shown). Importantly, the heightened inflammatory response to *Ft* mutants cannot be attributed to greater intramacrophage replication at 24 h (Figure 9E).

## Discussion

We have reported that both strains of *Ft* used (*i.e.*, LVS and SchuS4), when adapted to their mammalian host environment, rapidly (within hours) elicit a TLR2- and NF-κB-dependent response in the lungs that is principally anti-inflammatory in nature. This anti-inflammatory response is typified by production of IL-10 and TGF-β and the development and proliferation of tDCs and T_regs_ [21]. Within such a cellular milieu rich in neutrophils, but lacking in TNF, IL-1β, and IL-6, *Ft* replicate nearly unfettered in what would normally be an inhospitable environment. It is not until 72 h or later that key T_H_1-oriented pro-inflammatory cytokines are produced at substantial levels and clearance of bacteria from tissues ensues.

While a variety of *in vitro* work using non-HAd, MHB-grown *Ft* implicate TLR2 in mediating the release of a variety of cytokines by mouse and human monocytes/macrophages, select pro-inflammatory mediators are absent during the first 3 days of pulmonary infection in mice. Since *Ft* is incapable of eliciting classical T_H_1-oreinted pro-inflammatory cytokines during early-phase disease, we evaluated whether the bacteria become inherently more pro-inflammatory during the course of infection. This was found not to be the case as *Ft* recovered from highly inflamed lung tissue 6 days p.i. were incapable of eliciting a pro-inflammatory response from BMDMs comparable to that triggered *in vitro* by non-HAd, MHB-grown *Ft*. Consider also that there are plenty of ‘free’ bacteria in the bloodstream during late-phase disease [27] and yet, only low levels of select pro-inflammatory cytokines are present in the serum. Nor could the discrepancy between *in vitro* and *in vivo* findings be attributed to changes in host cell responsiveness to *Ft* during the course of infection.

Another important point to consider is that the present study and other recent reports [16], [17], [21], [24] clearly demonstrate that the choice of medium in which *Ft* LVS and SchuS4 are cultivated has a profound impact on whether *in vitro* host cell responses reflect those observed during mammalian infection. In contrast to HAd-*Ft*, the non-HAd-*Ft* elicited significant amounts of select pro-inflammatory cytokines from various cell types, irrespective of whether the cells were murine-derived or human in origin. The *in vitro* production of these select pro-inflammatory cytokines was dependent on CD14 and TLR2. Experiments failed to reveal a role for any other mouse or human TLRs including TLR9, whose ligand is bacterial DNA. Recently, several studies have implicated a cytosolic DNA sensor, AIM2, in *Ft*-induced activation of caspase-1 and subsequent secretion of mature IL-1β [10]–[13]. Notably, all the aforementioned studies used *F. novicida* as well as *Ft* LVS and SchuS4 grown using MHB or scraped off MH-based chocolate agar plates. Confirming these findings, it was observed that non-HAd-*Ft* and isolated *Ft* DNA were capable of eliciting not only IL-1β, but also TNF and IL-6 in an AIM2-dependent and -independent manner. Neither *Ft* LVS nor SchuS4 DNA could provoke a T_H_1-oriented pro-inflammatory cytokine response in the absence of the transfection reagents DOTAP or Lipofectamine™ 2000. These results demonstrate the importance of delivery of agonist into the endocytic pathway as well as cytosol of the cell [23]. Left unanswered is why isolated *Ft* DNA, when appropriately localized, stimulates a response through AIM2, but not TLR9. Unlike non-HAd-*Ft*, HAd-*Ft* that also escape into and replicate within the cytosol fail to elicit IL-1β despite the presence of AIM2 and other DNA sensors. This paradox implies that a profound difference between MHB- and BHIB-grown *Ft* exists with regard to their ability to engage DNA sensors. Both HAd- and non-HAd-*Ft* signals through TLR2/1/6 thus evoking an NF-κB-dependent extracytosolic response. However, when cytosolic, only the non-HAd-*Ft* is capable of engaging DNA-sensors (*e.g.*, AIM2) and triggering the production of TNF, IL-1β, and IL-6; a DNase I-sensitive intracytosolic response. This observation was quite unexpected and interesting given that DNase I treatment did not kill the bacteria nor did it impair their ability to invade and replicate within cells. The possibility that bacteria released host cell-stimulatory proteins or nucleic acids into the growth media also was ruled out.

The role of extracellular bacterial DNA as a major pro-inflammatory component was recently reported for *Pseudomonas aeruginosa* biofilms [28]. Extracellular DNA can be secreted from bacteria via the type IV secretion pathway (which is not present in *Ft*) or by release through lysis of a fraction of the bacterial population [29]. This latter mechanism also is operative in the context of host cell infection by mutant strains of *F. novicida* [26]. These findings prompted us to investigate the overall structural integrity of *Ft* cultured in different growth media. Live/dead staining of the non-HAd and HAd-*Ft* revealed that ∼five-fold more bacteria grown in MHB were structurally-compromised as compared to their BHIB-grown counterparts. Damaged *Ft* is permeable to the ∼600 Dalton SYTOX® Green dye molecule possibly allowing its traversal across both the outer and inner bacterial membranes to bind its DNA ligand. The “porous” nature of *Ft* grown in MHB was confirmed by observing immune reactivity for *Ft* 50S ribosomal protein using a mAb. This same mAb showed no reactivity towards HAd-*Ft* even at the lowest dilution tested. Thus, cultivation of *Ft* in MHB exposes bacterial DNA as well as other internal components to the extracellular environment (and host cells in the vicinity), which may explain the *in vitro* capacity of non-HAd-*Ft* to elicit the select pro-inflammatory cytokines reported by our group [5] as well as many others [2]–[4].

Either alternatively or additionally, SYTOX® Green-positive *Ft* may be more prone to bacteriolysis in the cytosol as Peng *et al.* observed for *F. novicida* mutants [26]. We report here that many of the same mutations in *Ft* LVS and SchuS4 confer an aberrant pro-inflammatory phenotype on the bacterium, despite the diverse functions of the gene products or host-adaptation through growth in BHIB. Another contributing factor underlying the difference in the pro-inflammatory character of non-HAd versus HAd-*Ft* is that the former express significantly less capsule on their surface thus facilitating bacterial recognition by effectors of innate and adaptive immunity [24]. Importantly, an inverse relationship exists between the pro-inflammatory potential of non-HAd-*Ft* and a variety of mutants and their virulence in mouse models of infection. We postulate that wild-type and mutant non-HAd-*Ft* and even HAd-*Ft* mutants are severely attenuated not by virtue of an inability to suppress NF-κB-mediated host immunity, but because they aberrantly elicit TNF, IL-1β, IL-6, IL-12, and IFN-γ, immune modulators associated with ‘sterilizing’ innate responses to infection. It is worth noting that during natural infection with *Ft* LVS a significant log-scale decrease in bacterial burden in lung, liver, and spleen is not observed until after day 3, coincident with the elaboration of the select T_H_1-oriented cytokines absent during early-phase disease. Though such experiments are complicated by the extreme virulence of the strain, whether a comparable inverse relationship between onset of T_H_1-oriented cytokine production and clearance of SchuS4 exists is under investigation.

In summary, the findings described above lend considerable support to the notion that *Ft* senses its environment and may adjust its pro-inflammatory potential accordingly. This immune evasive strategy is not qualitatively dissimilar to the temperature-induced alteration in LPS structure that imparts a hypo-inflammatory phenotype on *Yersinia pestis* [30]. However, instead of the site of inoculation being immunologically “silent” immediately after infection the presence of *Ft* in the lung elicits recruitment of neutrophils and macrophages and establishes an anti-inflammatory milieu in which bacteria replicate unencumbered by potent antimicrobial innate immune responses (*i.e.*, production of TNF, IL-1β, IL-6, IL-12p70, and IFN-γ). The ability of non-HAd-*Ft* to elicit these select T_H_1-oriented cytokines *in vitro* reflects aberrant recognition of bacterial DNA by myeloid cells and is inconsistent with what is observed during infection. Regarding initiation of murine infection with non-HAd vs. HAd-*Ft*, neither elicits select T_H_1-oriented cytokines during early-phase (day 2) disease owing to rapid adaptation of MHB-grown *Ft* to its new mammalian environment. However, the time it takes for this host adaptation to occur (∼12–16 h) is reflected in a difference in MTD [20], [24]. Finally, this body of work should stimulate re-evaluation of the field's understanding of mechanisms underlying *in vitro Ft*-host cell interactions as they relate to tularemia pathogenesis.

## Methods

### Ethics statement

Food and water were provided to mice *ad libitum* and this study was carried out in strict accordance with the recommendations in the Guide for the Care and Use of Laboratory Animals of the National Institutes of Health. The protocols (#12-04002 and 12-04003) were approved by the Institutional Animal Care and Use Committee of Albany Medical College.

### Animals

C3H/HeN mice (Taconic Farms, Germantown, NY), C57BL/6 mice (purchased from National Cancer Institute), C3H/HeN CD14^−/−^, C3H/HeN TLR2^−/−^, and C57BL/6 TLR2^−/−^ mice were housed in the Animal Resources Facility at Albany Medical College.

### Bacteria

*Ft* LVS was kindly provided by Dr. Karen Elkins (U.S. FDA, Bethesda, MD). *Ft* SchuS4 was obtained from the USAMRIID (Frederick, MD). Bacteria were cultured in MHB or BHIB or isolated from infected BMDMs as previously described [21]. Briefly, a single colony picked from a MHB-agar plate was used to initiate a 5 ml MHB or BHIB culture that was maintained for 12 h at 37°C while shaking at 220 RPM. These ‘starter’ cultures then were used to inoculate (1∶200) a 100 ml MHB or BHI culture that was maintained for 12–16 h. Bacteria were harvested when cultures achieved an early log-phase A_600 nm_ optical density of 0.2 at which point colony forming units (CFU) per ml were determined by serial dilution and colony plating. Both fresh bacterial cultures and frozen aliquots stored in liquid nitrogen elicited identical responses when used in *in vitro* and *in vivo* studies.

### Animal infection

Mice were infected and survival experiments were performed as previously described [5]. BALF and serum samples were collected and stored at −20°C. Organs were excised for histological evaluation, to determine bacterial burden, measure cytokine/chemokine levels, and isolate cells for flow cytometric analysis as previously described [5], [21].

### Luciferase reporter assay

HEK293 cells were transfected with the indicated TLRs plus 5X-NF-κB-luciferase (Stratagene, La Jolla CA) and empty vector to equal 200 ng/well of total DNA. After overnight culture in DMEM containing L-Gutamine (4 mM), glucose (0.45%), sodium pyruvate and supplemented with 1% HEPES and 10% heat-inactivated FBS (*i.e.*, cell culture medium), cells were treated with various stimuli for 24 h. Cells were exposed to control agonist or *Ft* at a multiplicity of infection (MOI) of 0.1 or 1 that represented 2 µg/ml or 20 µg/ml total *Ft* proteins, respectively. HEK293 cells expressing mouse and human TLR9 were incubated for 24 h with control CpG and *Ft* LVS DNA. Cell lysates were assayed for luciferase activity using the Luciferase Reporter Assay System (Promega Corp., Madison, WI).

### Isolation of murine BMDMs, generation of murine BMD-DCs, immortalized murine AIM2^−/−^ BMDMs, and human macrophages

BM cells were isolated to enrich for BMDMs and to generate BMD-DCs as previously described [21]. Primary human monocytes were purchased from the University of Nebraska (Lincoln, NE) and cultured as previously described [31]. The AIM2^−/−^ macrophage cell line was generated by infecting primary bone marrow cells with J2 recombinant retrovirus (a kind gift from Dr. Katherine Fitzgerald) as described previously [32]. For some experiments wild-type and TLR9^−/−^ or AIM2^−/−^ BMDMs were incubated with *Ft* DNA in the presence or absence of DOTAP (Roche Applied Science, Indianapolis, IN) or Lipofectamine™ 2000 (Invitrogen Life Technologies, Carlsbad, CA) used at 15 µg or 10 µg per well, respectively. Cell culture supernatants were collected at 24 h p.i. and analyzed for the presence of cytokines/chemokines as described below.

### DNase I treatment of bacterial DNA and whole live bacteria

Genomic DNA (8 µg/ml) purified from *Ft* LVS or live non-HAd bacteria (5×10^7^ CFU/ml) was either left untreated or incubated with 10 units of recombinant RNase-free, DNase I (Roche Applied Science, Mannheim, Germany) for 15 minutes at 37°C. For genomic DNA, the reaction was stopped by heating the mixture at 72°C for 10 min. For live non-HAd *Ft*, the bacteria were pelleted by microcentrifugation, washed twice with cell culture medium and thereafter were used for infection of wild-type and AIM2^−/−^ BMDMs. For the results presented in Figure 3F, frozen stocks of non-HAd *Ft* either were directly placed on wild-type and AIM2^−/−^ BMDMs at a MOI of 100 or were first pelleted by microcentrifugation, washed twice with and resuspended in cell culture medium prior to infection of the BMDMs.

### Cytokine measurements

Lung homogenates and cell culture supernatants were assayed for a variety of cytokines and chemokines. Mouse Inflammation Cytometric Bead Array kit or individual Flexsets (BD Pharmingen, San Diego, CA) were used for measurement of cytokine and chemokine levels by flow cytometry as described elsewhere [21].

### Evaluation of the structural integrity of HAd- and non-HAd-*Ft*

The structural integrity of bacteria grown to an early log-phase A_600 nm_ optical density of 0.2 was evaluated using SYTOX® Green nucleic acid stain (Invitrogen Life Technologies). Flow cytometry was performed on stained bacteria utilizing an LSR II instrument (Becton Dickinson, Franklin Lakes, NJ). In addition, *Ft* LVS cultivated in MHB or BHIB were used for coating 96 well microtiter plates (Corning Life Sciences, Lowell, MA) at 5×10^6^ CFU/well. Plates were coated for 2 h at 37°C with whole bacteria or lysates, then washed with PBS containing 0.05% Tween-20, and blocked overnight at 4°C with 10% horse serum in PBS. Blocked plates were incubated with serially diluted normal mouse sera, immune mouse sera, or primary antibody (AB10) directed against *Ft* 50S ribosomal protein for 90 min. at 37°C. After washing, plates were incubated with anti-mouse IgG secondary mAb (1∶5000) (Invitrogen Life Technologies) for 60 min, at 37°C. After washing, Streptavidin-HRP (125 ng/ml) (Invitrogen Life Technologies) was added to the wells and the plates were allowed to incubate at 37°C for 20 minutes. The plates were washed and developed with 100 µl TMB substrate/well for 10 min at 37°C. The reaction was stopped with100 µl 1 N H_2_S0_4_, and the plates were read at 450 nM.

### Intramacrophage replication assay

Intramacrophage replication of bacteria was evaluated as previously described [5]. Briefly, primary BMDMs (2.5×10^5^ cells/well) were infected with wild-type and mutant HAd-*Ft* at a MOI of 100 for 2 h followed by gentamycin (50 µg/ml) treatment for 2 h to kill extracellular bacteria. Cells were washed, incubated for the indicated periods of time, and then lysed with 200 µl of 0.1% filter sterilized sodium deoxycholate. Both supernatant and lysate suspension were combined and centrifuged to recover a bacterial pellet. Bacteria were resuspended in BHIB and enumerated by colony counting.

### Isolation of bacteria from lung tissue

Lungs were harvested from mice at day 2 and 6 p.i. and were enzymatically-digested in FA buffer (BD, Franklin Lakes, NJ) containing 3.33 mg/ml of Type I collagenase (Worthington Biochemical Corp., Lakewood, NJ) for 1 h at 37°C. Digested tissue was passed through a cell strainer and intact cells were separated from the supernatant by centrifugation at 200×*g* for 10 min. Intracellular bacteria were collected by repetitive passage of the cells through a 1 cc syringe fitted with a 25-gauge needle. Extracellular bacteria were recovered from supernatants by microcentrifugation at 10,000 RPM for 20 min. and were combined with the intracellular bacterial fraction.

### Statistical analysis

All results were expressed as mean ± SEM from two or more independent experiments. Depending upon the distribution of the data set, comparisons between groups were made using a parametric ANOVA test with Bonferroni's post-test or a nonparametric Kruskal-Wallis test with Dunn's post-test. Student's t-test was applied where indicated. Differences between control and experimental groups were considered significant at α = 0.05 level.

## Supporting Information

Figure S1 **DNase I treatment of DNA ablates TNF and IL-6 release from wild-type and AIM2^−/−^ BMDMs.** (A) Wild-type and (B) AIM2^−/−^ BMDMs were incubated either in the absence or presence of DOTAP with 8 µg/ml of *Ft* LVS genomic DNA. DNA was either untreated or incubated with DNase I. Supernatants collected after 24 h were analyzed for the presence of cytokines by CBA. **P<0.01 and ***P<0.001. (C) & (D) Similar experiment as in (A) & (B) were conducted using Lipofectamine™ 2000 as the transfection agent. ***P<0.001. (All results shown were subjected to One-way ANOVA with Bonferroni's Post-test). (E) & (F) Wild-type and AIM2^−/−^ BMDMs were infected with *Ft* LVS cultivated in MHB either untreated or treated with DNase I at a MOI of 100. Supernatants collected after 24 h were analyzed for the presence of cytokines by CBA. *P<0.01, **P<0.01, ***P<0.001.
(DOCX)

Figure S2 **HAd-** ***Ft*** **LVS fails to elicit T_h_1-type pro-inflammatory cytokines from primary human peripheral blood-derived macrophages.** Human peripheral blood-derived monocytes (5×10^5^ cells/well) were infected at a MOI of 100 with *Ft* grown in MHB or BHIB or recovered from MΦ. Supernatants were collected after 24 h and analyzed for the presence of IL-6 (A), IL-8 (B), IL-10 (C), and IFN-γ (D) by CBA. Results represent the mean ± SEM from two independent experiments. **P<0.01 and ***P<0.001. (One-way ANOVA with Bonferroni's Post-test).
(DOCX)
