# Supplementary figures and images for: Discordant Results Obtained with Francisella tularensis during In Vitro and In Vivo Immunological Studies Are Attributable to Compromised Bacterial Structural Integrity
Source: PLoS One. 2013 Mar 12;8(3):e58513. doi: 10.1371/journal.pone.0058513 (PMC3595284; doi:10.1371/journal.pone.0058513)

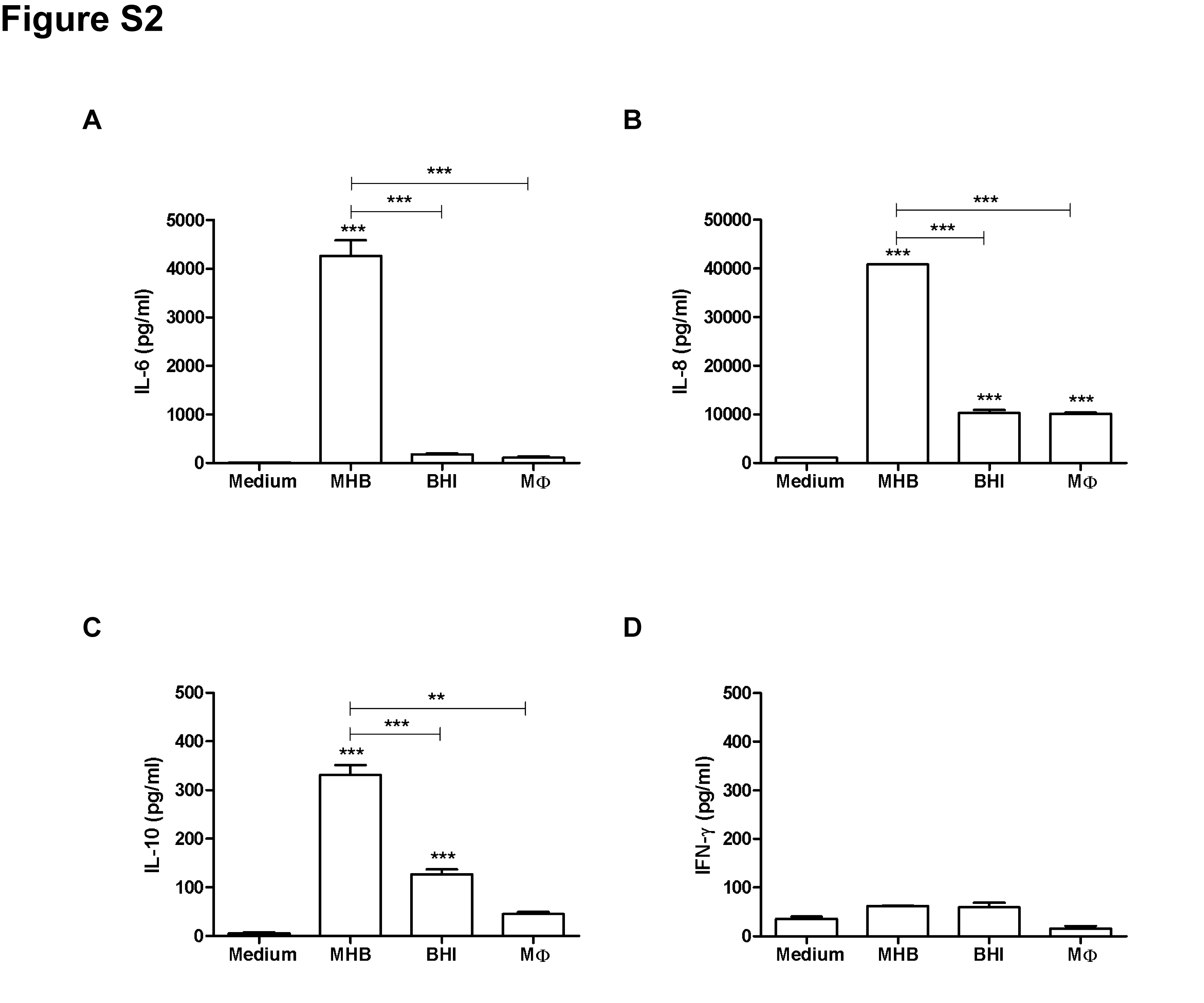

Supplement: Figure S2 — HAd- Ft LVS fails to elicit Th1-type pro-inflammatory cytokines from primary human peripheral blood-derived macrophages. Human peripheral blood-derived monocytes (5×10^5^ cells/well) were infected at a MOI of 100 with Ft grown in MHB or BHIB or recovered from MΦ. Supernatants were collected after 24 h and analyzed for the presence of IL-6 (A), IL-8 (B), IL-10 (C), and IFN-γ (D) by CBA. Results represent the mean ± SEM from two independent experiments. **P<0.01 and ***P<0.001. (One-way ANOVA with Bonferroni's Post-test). (DOCX) [file pone.0058513.s002.docx]
