# Supplementary material for: Discordant Results Obtained with Francisella tularensis during In Vitro and In Vivo Immunological Studies Are Attributable to Compromised Bacterial Structural Integrity
Source: PLoS One. 2013 Mar 12;8(3):e58513. doi: 10.1371/journal.pone.0058513 (PMC3595284; doi:10.1371/journal.pone.0058513)
Supplement: Supplementary file 2 [file pone.0058513.s001.docx]

Figure S1

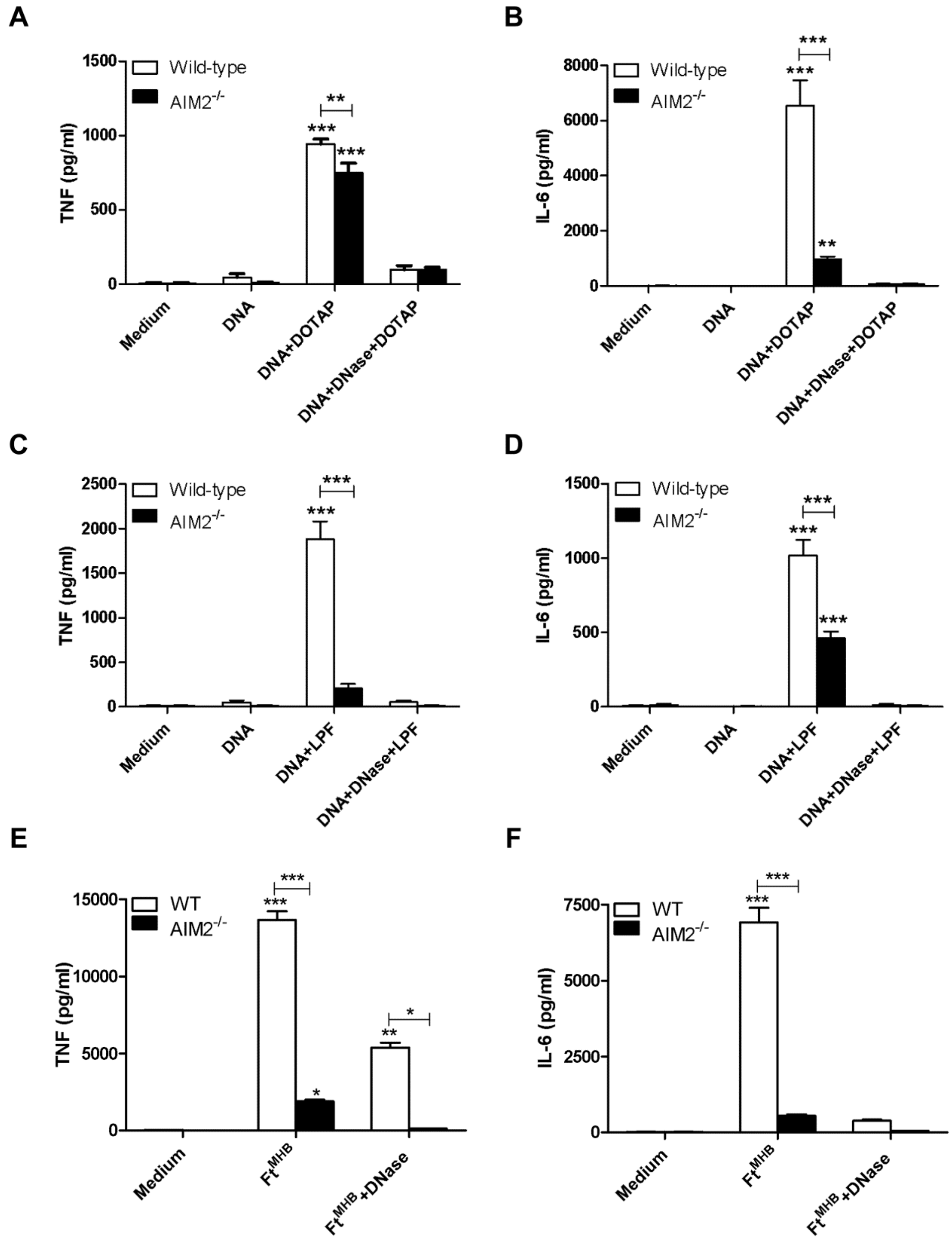

A
Wild-type
AIM2-/-
TNF (pg/ml)
Medium
DNA
DNA+DOTAP
DNA+DNase+DOTAP
B
IL-6 (pg/ml)
C
DNA+LPF
DNA+DNase+LPF
D
E
WT
Ft^MHB
Ft^MHB+DNase
F
